# The unity of opposites: Strategic interplay between bacterial effectors to regulate cellular homeostasis

**DOI:** 10.1016/j.jbc.2021.101340

**Published:** 2021-10-23

**Authors:** Shalini Iyer, Chittaranjan Das

**Affiliations:** 1 Department of Chemistry, Purdue University, West Lafayette, Indiana, USA

**Keywords:** protein structure, bacteria, protein–protein interaction, membrane trafficking, ubiquitylation (ubiquitination), ligases, posttranslational modification (PTM), *Legionella*, manipulation, Rab GTPases, ARD, ankyrin repeat domain, ATase, adenylyl-transferase, CaM, calmodulin, CMP, cytidine monophosphate, DUB, deubiquitinase, GAP, GTP-hydrolysis-activating protein, GDI, guanine dissociation inhibitor, GEF, guanine-nucleotide exchange factor, GS-ATase, glutamine synthetase-adenylyltransferase, IDTS, Icm/Dot translocated substrate, LCV, *Legionella*-containing vacuole, mTORC1, mammalian target of rapamycin complex 1, NAD, nicotinamide adenine dinucleotide, P4M, phosphatidylinositol-4-phosphate binding domain of SidM, PR, phospho-ribose, PTM, posttranslational modification, T4SS, type IV secretion system, Ub, ubiquitin, Ub-ADPR, ADP-ribosylated Ub

## Abstract

*Legionella pneumophila* is a facultative intracellular pathogen that uses the Dot/Icm Type IV secretion system (T4SS) to translocate many effectors into its host and establish a safe, replicative lifestyle. The bacteria, once phagocytosed, reside in a vacuolar structure known as the *Legionella*-containing vacuole (LCV) within the host cells and rapidly subvert organelle trafficking events, block inflammatory responses, hijack the host ubiquitination system, and abolish apoptotic signaling. This arsenal of translocated effectors can manipulate the host factors in a multitude of different ways. These proteins also contribute to bacterial virulence by positively or negatively regulating the activity of one another. Such effector–effector interactions, direct and indirect, provide the delicate balance required to maintain cellular homeostasis while establishing itself within the host. This review summarizes the recent progress in our knowledge of the structure–function relationship and biochemical mechanisms of select effector pairs from *Legionella* that work in opposition to one another, while highlighting the diversity of biochemical means adopted by this intracellular pathogen to establish a replicative niche within host cells.

Bacterial secretion systems are complex cellular machines used to translocate toxins and virulence factors into host cells. Nine major secretion systems (Types I–IX) have been described in Gram-negative and Gram-positive bacteria. Specialized systems, such as type III and type IV secretion apparatus, have been central to the evolution of many intracellular pathogenic Gram-negative bacteria (1, 2, 3). These pathogens produce many proteins called effectors that differ from bacterial toxins in that they do not irreversibly disrupt the cellular equilibrium of their host (4). Instead, they help create a facultative niche for the pathogen's survival by functioning in concert with each other and subtly manipulating critical cellular pathways of the infected eukaryote (5, 6). Pathogenic bacteria such as *Legionella pneumophila* exploit eukaryotic cell functions and influence multiple signaling events by translocating over 330 effectors *via* its Type IV secretion system, also known as the Icm/Dot transporter (7, 8, 9, 10, 11). These effectors, collectively termed Icm/Dot translocated substrates (IDTS), remain cytosolic, localize to the LCV, or traffic to different organelles.

Bacterial effectors are commonly known to mimic the activities of eukaryotic proteins despite lacking significant similarity in amino acid sequence with the host proteins (12). For example, the bacterial effector SopE from *Salmonella typhimurium* functions as a guanine-nucleotide exchange factor (GEF) targeting the Rho-family of GTPases (13) while sharing little sequence homology with eukaryotic enzymes of the same function. Another common theme seen among bacterial effectors is redundancy, which extends beyond gene duplication. The most well-recognized form is functional redundancy, whereby two effectors may catalyze the same reaction and have similar substrate specificities, thus allowing one to compensate the absence of the other, as exemplified by the SidE family proteins from *L. pneumophila* that catalyze ubiquitination of host proteins associated with the endoplasmic reticulum. Redundancy can also exist between unrelated bacterial proteins. For example, *Legionella* effectors such as SidM, AnkX, and SidE proteins are all known to target Rab1; however, the mechanisms by which each of these effectors modulates the Rab protein are entirely different, resulting in different modifications on the GTPase.

Among the diverse ways of interaction with their host, pathogens have evolved a variety of means of manipulating host pathways by targeting their posttranslational modifications (PTMs) (14, 15). PTMs can range from addition of relatively small chemical groups, such as acetyl, hydroxyl, phosphate, AMP, ADP-ribose, or phospholipids, to more complex forms involving conjugation of small proteins, such as ubiquitin (Ub) or ubiquitin-like proteins (Ubls), to other proteins. Modification of host targets *via* effector-mediated enzymatic activities allows intracellular bacteria to remodel cellular processes relatively quickly and reversibly, if needed. While most effector activities are usually directed against host proteins, it is becoming increasingly evident that pathogens have developed another layer of complexity by regulating effector–effector interactions. Such modulation occurs when the translocated bacterial proteins either indirectly counterbalance their activity in a shared host pathway or directly interact with one another to suppress or enhance the associated function.

*Legionella* represents one of the most elaborate cases of cross talk between host cellular processes and the effectors it translocates. This pathogen has become a popular model for understanding both the role of the effectors in infection and the affected host signaling mechanisms, examples of which have been covered extensively in several past reviews (16, 17, 18, 19, 20). In this review we focus on functionally antithetic effectors from *L. pneumophila* with well-established biological function. This review intends to provide structural and mechanistic insights into specific examples (Table 1) that best illustrate the existence of effectors carrying out opposing functions in this organism. These effectors seem to work in concert to help the bacteria establish a balanced lifestyle within the host while avoiding catastrophic effects on the host environment.

**Table 1:** A list of all effector-effector pairs discussed in this review

#	Effector 1	Effector 2	Biochemical activity		PDB IDs		
			Effector 1	Effector 2	Effector 1	Effector 2	Complex
1	MavC (lpg2147)	MvcA (lpg2148)	Ubiquitination of UbE2N	Deubiquitination of UbE2N	5TSC	5SUJ	6UMP, 6ULH, 6UMS, 6P5B and 6P5H, 6KL4, 6KFP, 7BXG, 6KG6, 6K11, 6JKY
2	MavC (lpg2147) MvcA (lpg2148)	lpg2149	Ubiquitination of UbE2N	Inhibits both MavC and MvcA by directly binding to the inhibitors		5DPO	6K3B
			Deubiquitination of UbE2N				
3	SdeA (lpg2157) SdeB (lpg2156) SdeC (lpg2153) SidE (lpg0234)	SidJ (lpg2155)	Adds phosphoribosyl linked Ub to substrates	Glutamylates members of the SidE family and inhibits their activity	5YSJ, 5YSK, 6G0C, 5YIM, 5YIJ, 6B7Q, 5CRA, 5CRB, 5CRC, 5ZQ2	6OQQ, 6PLM, 6S5T, 6K4R, 6K4L, 6K4K	5YSI, 5YIK
4	SdeA (lpg2157)	SdeD (DupB) (lpg2509)	Adds phosphoribosyl linked Ub to substrates	Removes phosphoribosyl linked Ub from substrates		6B7P	6B7M, 6B7O
5	SidH (lpg2829)	LubX (lpg2830)		Functions as an E3 ubiquitin ligase to ubiquitinate SidH	-	4WZ0, 4WZ2, 4XI1	4WZ3
6	SidM (lpg2464)	SidD (lpg2465)	Ampylates Rab1-GTPase. Functions as a GEF/GDF for Rab1 GTPase	DeAMPylates Rab1-GTPase	3L0M, 3NKU, 4MXP, 3N6O, 3JZ9	4IIP, 4IIK,	3L0I, 2WWX, 3JZA, 5O74, 6YX5, 3TKL, 5O74
	SidM (lpg2464)	LepB (lpg2490)		Functions as a GAP for Rab1 GTPase		4I1M, 4JW1	4I1O, 4IRU, 4JVS
7	RavJ (lpg0944)	LegL1 (lpg0945)	Modular protein with an N-terminal papain-like cysteine protease fold and a C-terminal protein-protein interaction domain	Inhibits RavJ by blocking the enzyme’s catalytic site	4RXI, 4RXV, 4WRP	-	4XA9
8	AnkX (lpg0695)	Lem3 (lpg0696)	LegA8 (AnkX) catalyzes the transfer of phosphorylcholine to Ser76 of Rab1	A dephosphorylcholinase that reverses AnkX-mediated modification on Rab1	4BEP	-	4BER, 4BES, 4BET, 6SKU

The table provides the biochemical function carried out by each effector along with the associated PDB codes (where available).

## MavC and MvcA in atypical ubiquitination

**Figure 1:**
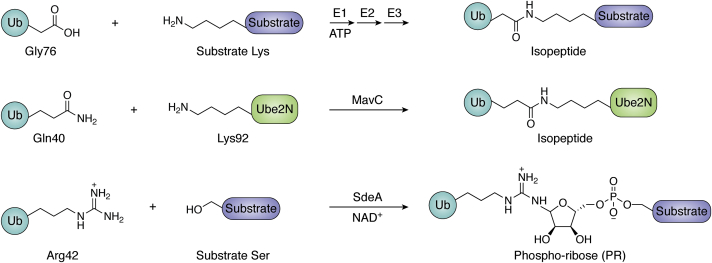
**Different modes of ubiquitination**.

Ubiquitination is one of the most widespread PTMs involved in almost every fundamental cellular process within eukaryotic cells (21). Ubiquitin is covalently attached to protein substrates *via* an isopeptide bond linking the last carboxylate of Ub (on Gly76) to the ε-amino group of lysine residues of substrates through the sequential actions of a trio of enzymes, an ATP-dependent Ub-activating E1 enzyme, a Ub-conjugating E2 enzyme, and an E3 Ub ligase (Fig. 1). This covalent attachment can be reversed when desired by deubiquitinases (DUBs) which catalyze the hydrolysis of the isopeptide bond. In addition to the vast array of other functions regulated by ubiquitination, it serves as the first line of defense against invading pathogens by mediating signaling events leading to innate immune response and xenophagy (22, 23, 24, 25). Accordingly, prokaryotic pathogens are often found to block (26, 27) and even manipulate the ubiquitination machinery to serve their purpose, sometimes using means outside the eukaryotic repertoire. In two recent examples, *L. pneumophila* was shown to ubiquitinate host targets by employing mechanisms independent of the classical three-enzyme system of eukaryotes: the SidE effectors use nicotinamide adenine dinucleotide (NAD^+^) to ubiquitinate serine residues of host targets *via* a phospho-ribose (PR) linker connecting the hydroxyl group with Arg42 of Ub (28, 29) (Fig. 1), whereas the MavC effector uses transglutaminase mechanism to cross-link Gln40 of Ub with a critical lysine residue (Lys92) of Ube2N (30), a reaction that does not even require a nucleotide cofactor (Fig. 1). These orthogonal modes of ubiquitination cannot be reversed by host DUBs, allowing the bacteria to remodel specific cellular pathways at will. However, aggressive manipulation of the Ub system and associated cellular processes can be detrimental to the pathogen. It could result in the host succumbing to the offense, ultimately limiting bacterial replication. Accordingly, *L. pneumophila* has evolved distinct strategies for a balanced control: in some cases, one effector switching off another to block its ubiquitinating activity, or, in other examples, one effector reversing the ubiquitin modification installed by another through a deubiquitinase-like reaction. In addition to these atypical ubiquitinating and deubiquitinating enzymes, *L. pneumophila* also possesses several effectors that mimic components of the host ubiquitination machinery, including the classical DUBs and E3 ligases, to co-opt the Ub system and interfere with Ub signals used in cellular defense (11, 31, 32, 33, 34, 35, 36).

**Figure 2:**
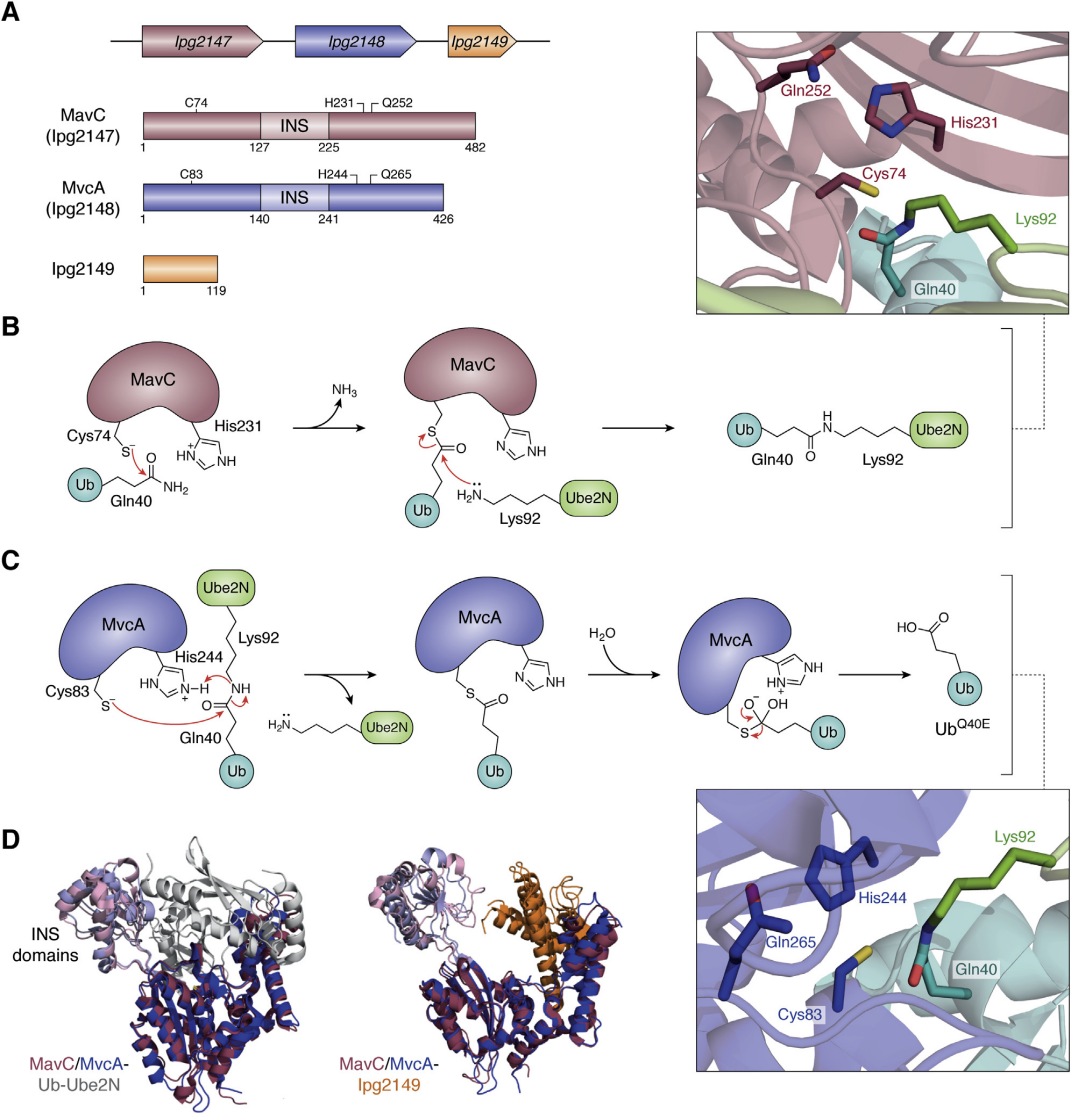
**MavC and MvcA in atypical ubiquitination.***A*, Orientation of the genes is indicated by the direction of the *arrows* they represent. Domains are labeled with the length of the proteins. Residues of the catalytic triad are shown. Also highlighted is the insertion (INS) domain in both MavC and MvcA. *B*, proposed mechanism of ubiquitination by MavC. In the first step, a thioester-linked acyl-enzyme intermediate is formed between the catalytic Cys74^MavC^ and Gln40^Ub^. In the second step, Lys92^Ube2N^ attacks the thioester intermediate resulting in an isopeptide linkage between Gln40^Ub^ and Lys92^UbE2N^. Arrangement of the catalytic residues in the active site is shown in a *boxed panel*. PDB codes: 5TSC, 6UMP, 6ULH, 6UMS, 6P5B, 6P5H, 6KL4, 6KFP, 7BXG, 6KG6. *C*, proposed mechanism of deubiquitination by MvcA. In the first step, the catalytic Cys83^MvcA^ attacks the isopeptide bond between Gln40^Ub^ and Lys92^UbE2N^ releasing Ube2N and forms a thioester-linked intermediate of MvcA with Ub. This is followed by deamidation of Ub (UbQ40E). Arrangement of the catalytic residues in the active site is shown in a *boxed panel*. PDB codes: 5SUJ, 6K11, 6JKY. *D*, structures of MavC and MvcA in complex with Ub-Ube2N and lpg2149. Overlay of the ribbon representations of the Ub-Ube2N (*gray*) and Lpg2149 (*orange*) complexes of MavC (*raspberry*) and MvcA (*blue*). The INS domains in both MavC and MvcA are highlighted by coloring them *lighter shades*. PDB codes: 5DPO, 7BXH, 7BXF, 6K3B.

MavC and its paralog MvcA are two effectors in *L. pneumophila* present on the same locus adjacent to each other with a 76-base pair intergenic space (Fig. 2*A*). These effectors were first described as cysteine-dependent deamidases based on their structural similarity to a group of bacterial deamidases of the Cif family, such as CHBP. They catalyze the conversion of Gln40 of Ub or the Ubl modifier NEDD8 to Glu40 (37, 38, 39). Subsequently, Gan *et al*. showed that MavC could catalyze monoubiquitination of Ube2N, a Ub-conjugating E2 enzyme essential for the synthesis of Lys63-linked polyubiquitin chains in the NFκB activation pathway (30, 40). Of the 40 different E2 enzymes encoded by eukaryotes, MavC specifically targets Ube2N through transglutaminase activity (Fig. 2*B*), ubiquitinating it (30) *via* an isopeptide crosslink between Gln40^Ub^ and Lys92^Ube2N^. This atypical ubiquitination at Lys92 renders the active site of the E2 enzyme inaccessible for its catalytic function of mediating Ub transfer from the E1 enzyme to the next recipient in the ubiquitination transfer cascade, resulting in blockade of Lys63-linked polyubiquitin chain synthesis (40).

MavC and MvcA share 52% sequence identity and are structurally very similar to one another (38, 39, 41). But unlike the Cif deamidases, MavC seems to have evolved to target Ube2N through the acquisition of a novel insertion domain (INS domain) that is absent in the deamidases. It is this insertion (INS) domain that enables MavC to carry out atypical ubiquitination of Ube2N (30, 41, 42, 43). The transglutaminase activity of MavC proceeds with, first, the formation of an acyl-enzyme thioester intermediate with Ub in which the carbonyl group of Gln40^Ub^ is linked to the S-atom of the catalytic thiol (Cys74^MavC^) (Fig. 2*B*). Formation of this intermediate is accompanied by release of ammonia, aided by His231^MavC^ acting as a proton donor to the leaving group. This intermediate is subsequently attacked by the ε−amino group of Lys92^Ube2N^ resulting in an isopeptide cross-link between Ub and the E2 target. The same intermediate is also prone to a nucleophilic attack by water, especially in the absence of the amine nucleophile, resulting in the deamidation of the glutamine side chain (41). However, the deamidase activity of MavC was not detected under infection conditions, suggesting that the transglutaminase activity could be its primary physiological function (30). Recently, four different groups have elucidated the three-dimensional structures of MavC in complex with its substrates and product (41, 44, 45, 46, 47). The structures revealed important insights into the role played by the INS domain in recruiting Ube2N and its conformational dynamics in promoting ubiquitination reaction over deamidation.

Cellular Ube2N exists as a heterodimer with Uev1a (48) while thioester linked to Ub through the catalytic cysteine (49, 50), a complex often referred to as the charged E2 complex. The charging of E2 (denoted by Ube2N∼Ub) occurs when Ub is transferred from E1 in the context of the E1-E2-E3 transfer cascade. Since the dimerization interface does not overlap with MavC binding, the Ub-charged heterodimer could be the actual physiological target of the effector. In that case, the transglutamination reaction likely occurs in an intramolecular fashion between Ub and Ube2N while being covalently tethered *via* the active-site thioester linkage in Ube2N∼Ub (41). The intramolecular reaction improves the probability of the transamidation reaction over the futile hydrolysis of the thioester intermediate that would otherwise result in Ub deamidation, which may cause a broader cellular impact, since the deamidated Ub derivative is substantially less useful in cellular ubiquitination events of the host (37). Specificity in recognition of Ube2N by MavC arises from interactions at the same interface on the E2 enzyme that is generally recognized by its cognate E3 ligases (51, 52). However, the MavC-Ube2N interaction is tighter than the interaction of Ube2N with its host E3-binding partners, such as TRAF6 (41, 53), allowing MavC to effectively engage its target amidst the host protein partners of Ube2N.

Remarkably, despite striking structural similarity with MavC, including identical catalytic residues and a similar INS domain (Fig. 2, *A* and *C*), MvcA catalyzes the removal of Ub from the ubiquitinated Ube2N (Ub-Ube2N), the product of the MavC-catalyzed modification. Cleavage of the isopeptide cross-link by MvcA leads to regeneration of native Ube2N in a reaction akin to cysteine-dependent deubiquitinase activity of eukaryotic DUBs (39, 47, 54). The thiol group of the catalytic cysteine of MvcA attacks the scissile isopeptide bond, forming a thioester intermediate with Ub as Ube2N leaves as the amine fragment (Fig. 2*C*). Hydrolysis of this intermediate releases Ub as the Q40E derivative. Thus, MvcA and MavC function similarly during the first step of their catalysis, forming the thioester intermediate with Ub accompanied by the departure of an amine group (ammonia in the case of MavC and Ube2N in the case of MvcA). The difference lies in the second step, wherein the MvcA catalysis involves water as the nucleophile in contrast to the Lys92^Ube2N^ amine nucleophile in the MavC reaction (Fig. 2, *B* and *C*). In addition to the same catalytic triad, contact regions between MvcA and its substrates also mimic those in the MavC-substrate complexes (Fig. 2*D*), pointing to an evolutionary adaptation that enables two enzymes sharing a common fold and mechanistic features to catalyze opposite reactions.

The MavC/MvcA pair provides a remarkable example of temporal regulation by *Legionella* necessitated by a specific requirement for Ub attachment and removal. In the initial stages of infection, MavC ubiquitinates Ube2N and dampens NF-κB signaling (55). However, the regular catalytic activity of Ube2N leading to NF-κB activation is beneficial to long-term intracellular growth of *L. pneumophila* even though its inhibition is necessary for blocking immune response in the early phase of infection (56). MvcA, on the other hand, is expressed ∼3 h postinfection (44), giving MavC ample time to blunt the immune response of the host while the bacteria are trying to establish a replicative niche within the host cell. The MavC-MvcA pair of effectors illustrate an important example of the subtle and precise interplay of bacterial effectors with specific host posttranslational pathways while avoiding systemic effects on a broad array of cellular processes.

Another gene sharing the same locus with *mavC* and *mvcA* is *lpg2149* (Fig. 2*A*) (37), separated from *mvcA* by an 88-base-pair intergenic space, suggesting that this separation might allow its independent expression and regulation (44, 57). Surprisingly, lpg2149 can inhibit both MavC and MvcA. Recently, the crystal structures of MavC and MvcA in complex with lpg2149 were elucidated (45, 46). The structures show that lpg2149 inhibits the enzymes by binding to a conserved structural element called the helical extension, thereby preventing Ub from binding (Fig. 2*D*). Thus, unlike MavC and MvcA, which have evolved to specifically inhibit Ube2N and restore it, respectively, at different time points during *Legionella* infection (30, 44), lpg2149 appears to possess a broader inhibitory activity toward both effectors through direct protein–protein interaction (37, 45). Gan *et al*. have shown that in a laboratory setting, lpg2149 expresses only in the early exponential phase and not in the early stages of *Legionella* infection, suggesting that lpg2149 exerts its inhibitory effects only when the bacteria have started to replicate (44). The significance of inhibition by lpg2149 remains unclear and requires further studies to understand the biological relevance of this multilayered regulation.

## SidM, SidD, and LepB: modulators of the Rab1 GTPase

The Rab family of small GTPases are critical mediators of eukaryotic endocytic and secretory vesicular trafficking events (58, 59, 60). Functioning as molecular switches, they cycle through two different nucleotide bound states to regulate protein–protein interactions necessary for vesicular trafficking events and other membrane-associated functions. The largely cytosolic, GDP-bound inactive Rab is activated by a guanine nucleotide exchange factor (GEF), which catalyzes the exchange of GDP for GTP to turn the protein to its active, membrane attached form. The GTP-bound Rab recruits specific protein partners to control docking and tethering steps between membrane compartments and cytoskeleton during vesicular trafficking events. The activated state of Rabs is temporally regulated by GTP-hydrolysis-activating proteins (GAPs), which promote GTP hydrolysis and return the Rab to its GDP-bound inactive form, which is subsequently extracted from the membrane by a guanine dissociation inhibitor (GDI) protein (61). Rab GTPases are targeted by intracellular bacteria, especially to bypass endocytic-lysosomal maturation of their phagosomes and subvert membrane trafficking from the endoplasmic reticulum (ER) (1, 59, 62, 63, 64).

Rab GTPases exploit their similar overall fold and conserved residues for nucleotide binding and catalysis while using individual structural differences in key variable regions to interact with specific binding partners, such as the GEFs and GAPs (59, 65). There are three essential recognition motifs in Rab variable regions: The P-loop (contacts the α and β-phosphates of the guanine nucleotide); Switch I (involved in Mg^2+^ coordination), and Switch II (consists of the DXXG motif that links binding of Mg^2+^ and the γ-phosphate of GTP). The inactive and active states of these GTPases are distinguished by the conformation of the switch loops (65), which along with the interswitch region, form interactions with almost all binding partners, including GEFs and GAPs. In the GTP-bound form, both Switch I and Switch II are held in place by interactions with the γ-phosphate group of GTP. Upon GTP hydrolysis, loss of these interactions and the release of the γ-phosphate group allow both switch regions to settle into their GDP-bound conformations.

**Figure 3:**
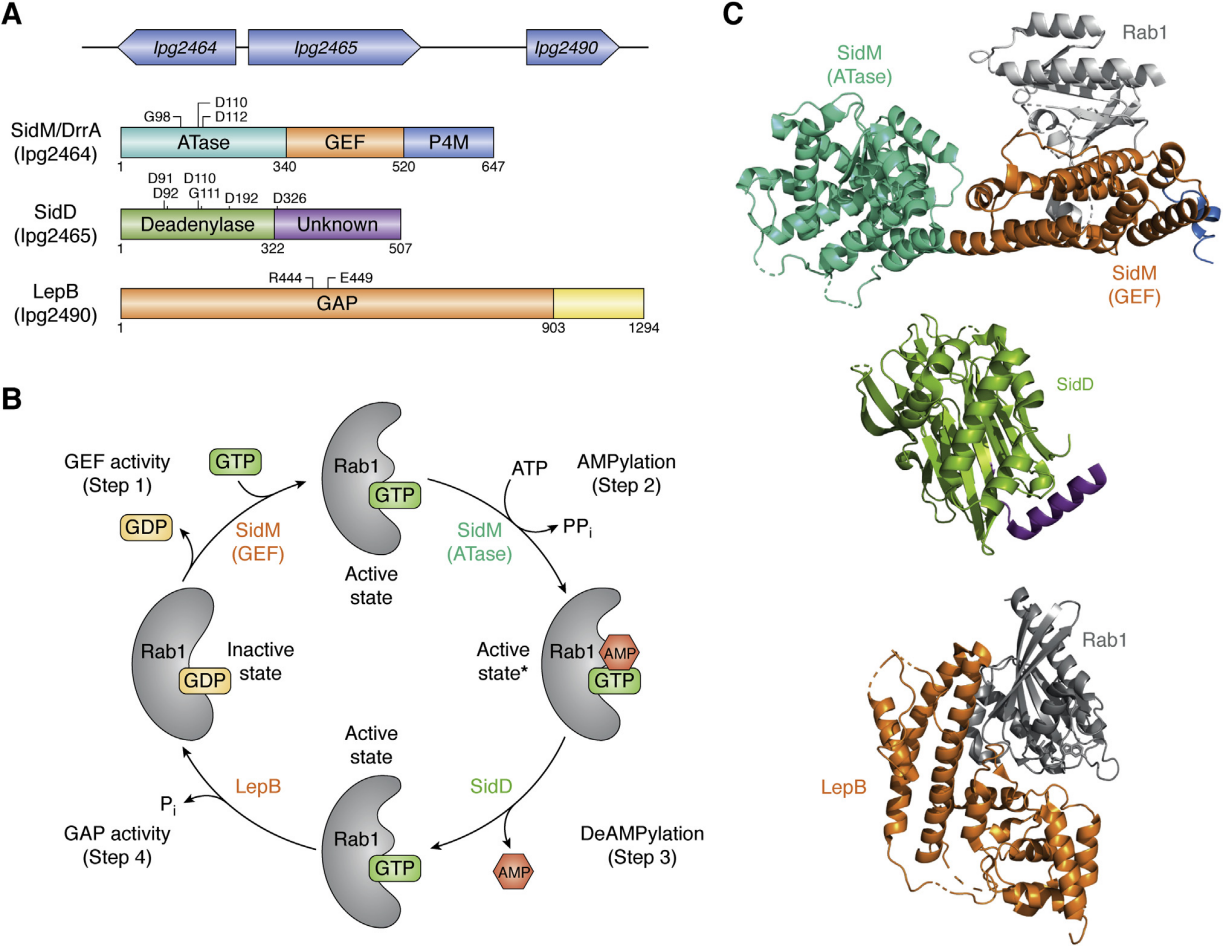
**SidM, SidD, and LepB: modulators of the Rab1 GTPase**. *A*, Orientation of the genes, shown in *blue*, is indicated by the direction of the *arrows* they represent. The direction of *sidM* is opposite to *sidD* and *lepB* in the locus. Domains are labeled with the length of the proteins. SidM: ATase, N-terminal adenylyltransferase domain (*cyangreen*); GEF, guanine-nucleotide exchange factor (*orange*); P4M, phosphatidylinositol-4-phosphate binding domain (*sky blue*); SidD: DeAMPylase, N-terminal deAMPylation domain (*green*) and a C-terminal domain of unknown function (*purple*); LepB: GAP, an N-terminal GTP-activating protein domain (*orange*) and a C-terminal domain of unknown function (*yellow*). Functionally important residues are shown. *B*, GDP-GTP exchange cycle for Rab1 involving SidM, SidD, and LepB. The GEF domain of SidM (*orange*) catalyzes the exchange of GDP to GTP (Step 1). The GTP-bound Rab1 (active state) is AMPylated (indicated by an *asterisk*) by the ATase domain of SidM (*cyangreen*) (Step 2). SidD (*green*) removes the AMP moiety from AMPylated-Rab1 (Step 3). The GAP domain of LepB (*orange*) inactivates Rab1 by hydrolyzing GTP to GDP (Step 4). *C*, crystal structures of SidM, SidD, and LepB. The structures are colored as per the scheme adopted for their domain architecture in Figure 3*A*.

One of the characteristic features of *Legionella* infection is the acquisition of an ER-like membrane coat on the LCV as the plasma-membrane-derived organelle matures into a phagosomal compartment supportive of bacterial replication (64, 66, 67). Among the several host proteins sequestered to the LCV is Rab1, a critical player during the initial stages of secretory pathways by promoting the transport and fusion of vesicles exiting the ER to the Golgi apparatus (68, 69), while also known to regulate membrane tethering events in autophagy (70). The recruitment of Rab1 is essential for further maturation of the LCV to a more ER-like organelle. SidM, SidD, and LepB are a unique trio of *L. pneumophila* effectors known to modulate Rab1 function to recruit membranes from the ER and the pre-Golgi intermediate compartment to the LCV (71, 72, 73). SidM is a multifaceted effector with functionally diverse domains (71, 74, 75). LepB neutralizes the different activities mediated by SidM (73, 76) and SidD (76, 77, 78) (Fig. 3*A*). Neunuebel *et al*. showed that translocation of SidM, SidD, and LepB is temporally regulated. Levels of SidM are high immediately following infection, commensurate with the recruitment of Rab1 to the LCV. Two hours postinfection, the levels of SidM start to decline as the levels of SidD and LepB rise, both of which are required to release Rab1 from the maturing LCV back to the host cytosol (79).

**Figure 4:**
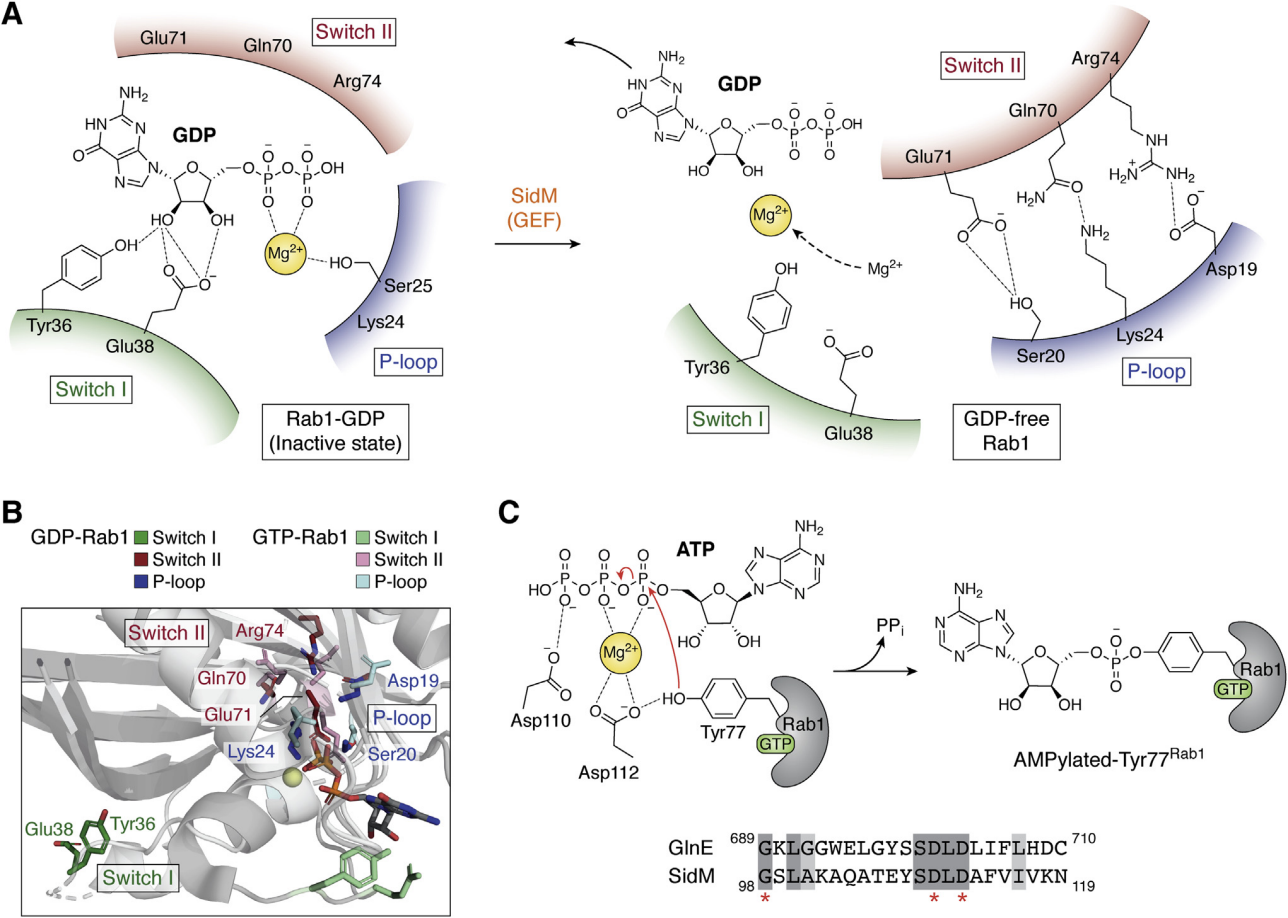
**Proposed catalytic mechanism ofSidM.***A*, SidM interaction with Rab1 leads to Switch I (*dark green*) of Rab1 to swing out. This causes Switch II (*maroon*) to interact with the P-loop (*dark blue*), pulling it inward. The resulting distortion of Switch I and II leads to the release of GDP. *B*, overlay of GDP-bound and GTP-bound Rab1. Ribbon representations of both the nucleotide-bound forms of Rab1 are shown in *gray*. Important residues at the binding interface are shown in stick representation. GDP-Rab1 residues from Switch I are shown in *dark green*, Switch II residues are shown in *maroon* and P-loop residues are shown in *dark blue*. Identical residues from the GTP-Rab1 structure are shown in *light green*, *pink*, and *light blue*, respectively. PDB codes: 3L0I, 2WWX, 3JZA, 5O74. *C*, proposed catalytic mechanism of Rab1 AMPylation by SidM. The catalytic aspartates (Asp110 and Asp112) attack the α−phosphate of ATP to attach the AMP group onto Tyr77^Rab1^*via* a phosphodiester bond. Also shown is a sequence alignment (BOXSHADE) of the GX_11_DXD motif from SidM and the GS-ATase (GlnE) from *E. coli* to highlight the sequence conservation of the active site residues.

SidM, also known as DrrA (defects in Rab1 recruitment protein A), features three distinct functional domains (Fig. 3*A*): an N-terminal adenylyl-transferase domain (ATase), a C-terminal lipid phosphatidylinositol-4-phosphate binding domain of SidM (P4M), and a central GEF domain (80). Once translocated into the host, SidM localizes to the LCV through membrane association mediated by its P4M domain (81), where it can act as both a GEF and a GDF (GDI-displacement factor) for Rab1 (80, 82, 83). It initiates Rab1 activation and subsequent LCV localization by first displacing Rab1 from the Rab1-GDI complex, followed by catalyzing the GDP to GTP exchange (Fig. 3*B*; Step 1). As far as GEF activity is concerned, Rab1 substrate specificity for SidM stems from interactions with residues in the Switch I loop (Asp34 to Ile41). Crystal structures show substantial conformational reorganization in the Rab1 switch regions upon SidM binding (Fig. 4, *A* and *B*). Although the mode of Rab1 activation is similar to that observed in eukaryotic GEFs, the GEF domain of SidM is structurally distinct from the eukaryotic GEFs (84) (Fig. 3*C*). Conformational changes during eukaryotic GEF-catalyzed nucleotide exchange involve structural rearrangements within the switch regions, with a more pronounced change in Switch I (Fig. 4*A*). When a GEF binds to the Switch I loop of the Rab GTPase, it destabilizes the interaction of the GTPase with the phosphate and the Mg^2+^ ion, pulling Switch I into an open conformation (Fig. 4, *A* and *B*). This destabilization also displaces the conserved Tyr36 (or Phe in some GTPases) from its interaction with the guanine nucleobase while causing the P-loop to lose its interactions with the phosphate groups of the nucleotide, thus lowering the affinity for GDP even further (59, 85, 86, 87, 88). Interestingly, the Switch I region in the SidM-Rab1 complex also disengages from the main body of Rab1 and rotates to face the opposite direction compared with its conformation in the Rab1-GDP and Ypt1-GDI complex (80, 84, 86). This rearrangement causes the guanosine binding site in Rab1 to distort, displacing Tyr36 and pushing Ser25 in the P-loop into the nucleotide-binding pocket, which induces Rab1 to adopt a more open conformation comparable to the other known GEF-Rab1 complexes, facilitating GDP release (Fig. 4, *A* and *B*). SidM binding also affects the Switch II region, stabilizing it in a conformation reminiscent of a GTP-bound Rab1 (89).

The N-terminal ATase domain of SidM further modulates the active state of Rab1 (Fig. 3*B*; Step 2) through the covalent attachment of an AMP moiety onto Tyr77 of Switch II (Fig. 4*C*) (75, 90), consequently locking Rab1 in its GTP-bound state. This AMPylation activity of SidM toward the GTP-bound Rab1 is nearly 270-fold higher than the GDP-bound form of Rab1, which implies that SidM preferentially targets active Rab1 (75) and AMPylation is preceded by the GEF function. SidM uses the classic GX_11_DXD motif in this reaction, where the aspartates coordinate the catalytic Mg^2+^ as seen in the *E. coli* glutamine synthetase-adenylyltransferase enzyme (GS-ATase) that catalyzes AMPylation of glutamine synthetase for regulating its activity (75, 91) (Fig. 4*C*). Two additional aspartates (Asp150^SidM^ and Asp249^SidM^) contribute to the binding of the Mg^2+^ ion, with Asp112^SidM^ serving as a general base to promote nucleophilic attack by the phenolic OH group of Tyr77^Rab1^ (75, 92) (Fig. 4*C*). Although the attachment makes little difference to the conformation of Rab1 and hence its GTP affinity, the modification of Tyr77 does prevent the *Legionella* GAP, LepB, or possibly host GAPs from binding to Rab1 prematurely (75, 90), thereby prolonging the lifetime of its activated state (90). Thus, the GEF and ATase domains of SidM appear to function collaboratively to extend retention of active Rab1 on the LCV membrane, at the same time thwarting the access of host GAPs to the GTPase.

**Figure 5:**
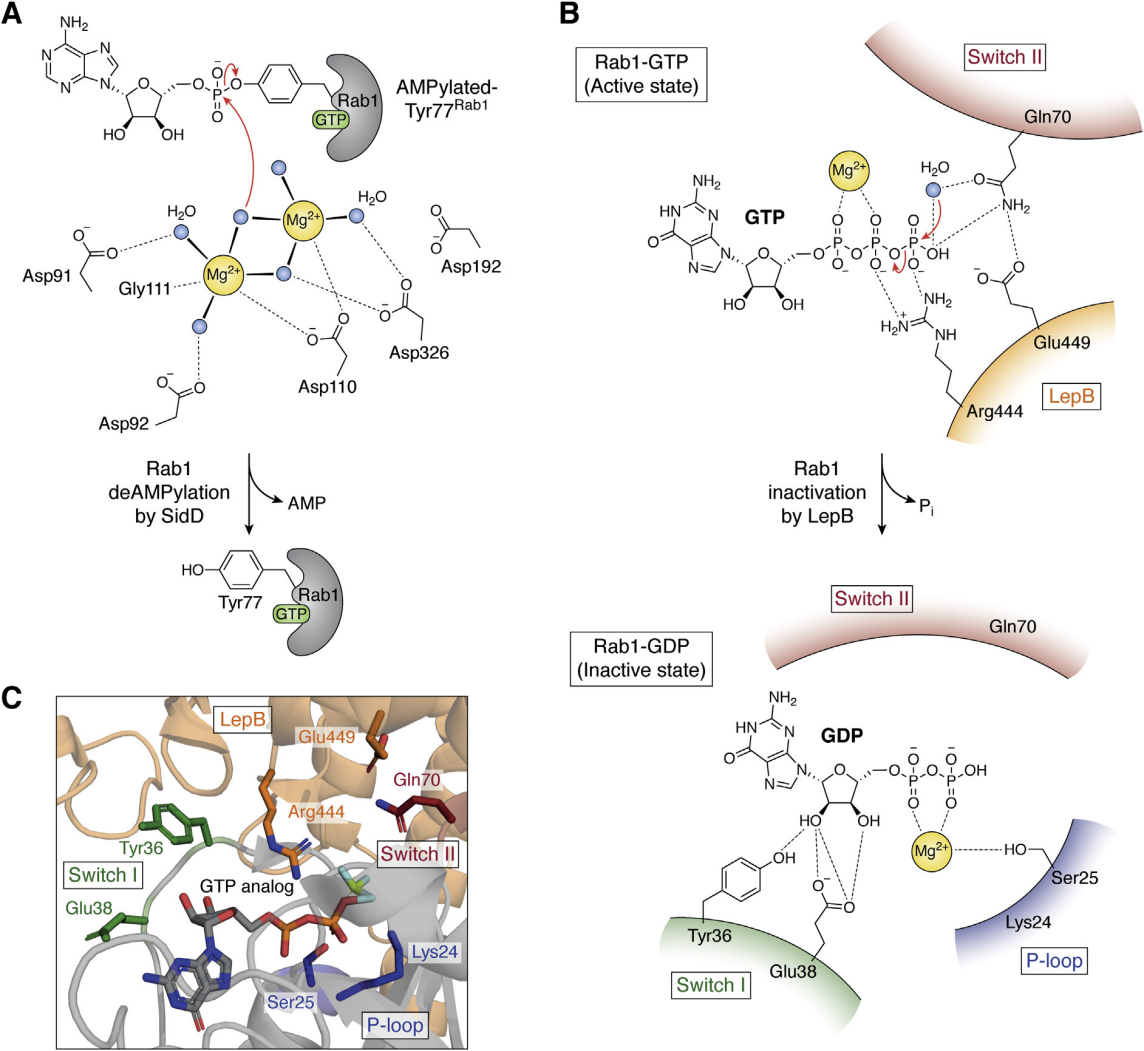
**Proposed catalytic mechanism of Rab1inactivation by SidD and LepB.***A*, De-AMPylation of Rab1 by SidD. The binuclear bridging water molecule acts as the nucleophile to cleave the adenylyl-O-tyrosyl linkage, thus freeing up Tyr77^Rab1^. PDB codes: 4IIP, 4IIK, 6RRE, 6RP4. *B*, proposed catalytic mechanism of Rab1 inactivation by LepB. The carbonyl of Gln70^Rab1^ (from the Switch II region) orients the catalytic water in the active site, causing it to attack the γ-phosphate of the GTP and convert it to GDP, thus inactivating Rab1. *C*, stick representation of the residues at the LepB-Rab1 binding interface. LepB residues are shown in *orange*. Rab1 residues from Switch I are shown in *green*, Switch II residues are shown in *maroon* and P-loop residues are shown in *blue*. PDB codes: 4I1O, 4IRU, 4JVS.

Overactivation and prolonged LCV retention of Rab1 may cause a drastic effect on host vesicular traffic events that rely on this critical ER-associated GTPase. SidD counteracts SidM-catalyzed AMPylation *via* its N-terminal enzymatic domain through deAMPylation activity (Fig. 3*B*; Step 3) while a putative membrane targeting segment in the C-terminal region may assist in the LCV localization (Fig. 3*A*) (76, 78, 93). The deAMPylase domain shares a distinct structural resemblance with a family of metal-dependent protein phosphatases (PPMs), such as human PP2Cα and bacterial PstP (94, 95). The catalytic pocket of SidD features a negatively charged region with two Mg^2+^ binding sites such as the binuclear metal-binding sites in PPMs (Fig. 5*A*). Five catalytic aspartates (Asp91, Asp92, Asp110, Asp112, and Asp326) and a binuclear bridging water, that acts as the nucleophile, coordinate the two metal ions. Hydrolysis of the adenylyl-O-tyrosyl linkage (AMP-Tyr77) releases AMP and restores Tyr77^Rab1^ to its unmodified form (Fig. 5*A*).

This deAMPylation frees up Rab1 from its continuous state of activation, thus allowing LepB to trigger GTP hydrolysis through its GAP activity (Fig. 3*B*; Step 4), leading to inactivation of Rab1 and their subsequent removal from the LCV. LepB is mechanistically homologous to the eukaryotic Rho/Ras-GAP rather than a Rab-GAP. Traditional Rab-GAPs (the so-called TBC GAPs) and some bacterial GAPs (of the VirA/EspG family) feature a catalytic glutamine finger in addition to the canonical arginine finger (Arg finger) (96, 97). LepB, however, features a glutamate residue (Glu449^LepB^) instead of the glutamine finger. Glu449^LepB^ occupies a structural position in apo-LepB equivalent to the canonical glutamine in the traditional Rab-GAPs (98, 99, 100). The catalytic Arg444 finger of LepB mediates a two-pronged polar interaction with the β-and γ-phosphates of the GTP (Fig. 5*B*). Upon binding to Rab1, Glu449^LepB^ undergoes a pronounced movement that triggers the side chain of Gln70^Rab1^ to flip toward the γ-phosphate of GTP. As a result, Gln70^Rab1^ adopts a similar position with respect to the γ-phosphate and a water molecule to what has previously been observed with the catalytic in-trans Gln finger (contributed by the substrate GTPase) in Ras-GAPs (Fig. 5*B*). The side chain of Gln70^Rab1^ would orient the water molecule for nucleophilic attack on the γ-phosphate center of GTP to facilitate its hydrolysis much like the catalytic in-cis Gln finger (contributed by the GAP) of TBC-like Rab GAPs (98, 99, 100) (Fig. 5*C*). Thus, LepB seems to employ the same sort of substrate-assisted catalysis commonly observed in Ras GAP-like catalytic mechanisms (98, 99, 100). Perhaps, a Ras GAP-like mechanism confers certain advantages for better kinetic control of Rab1 dynamics over the host GAP.

## AnkX and Lem3: parallel modulators of Rab1 function

**Figure 6:**
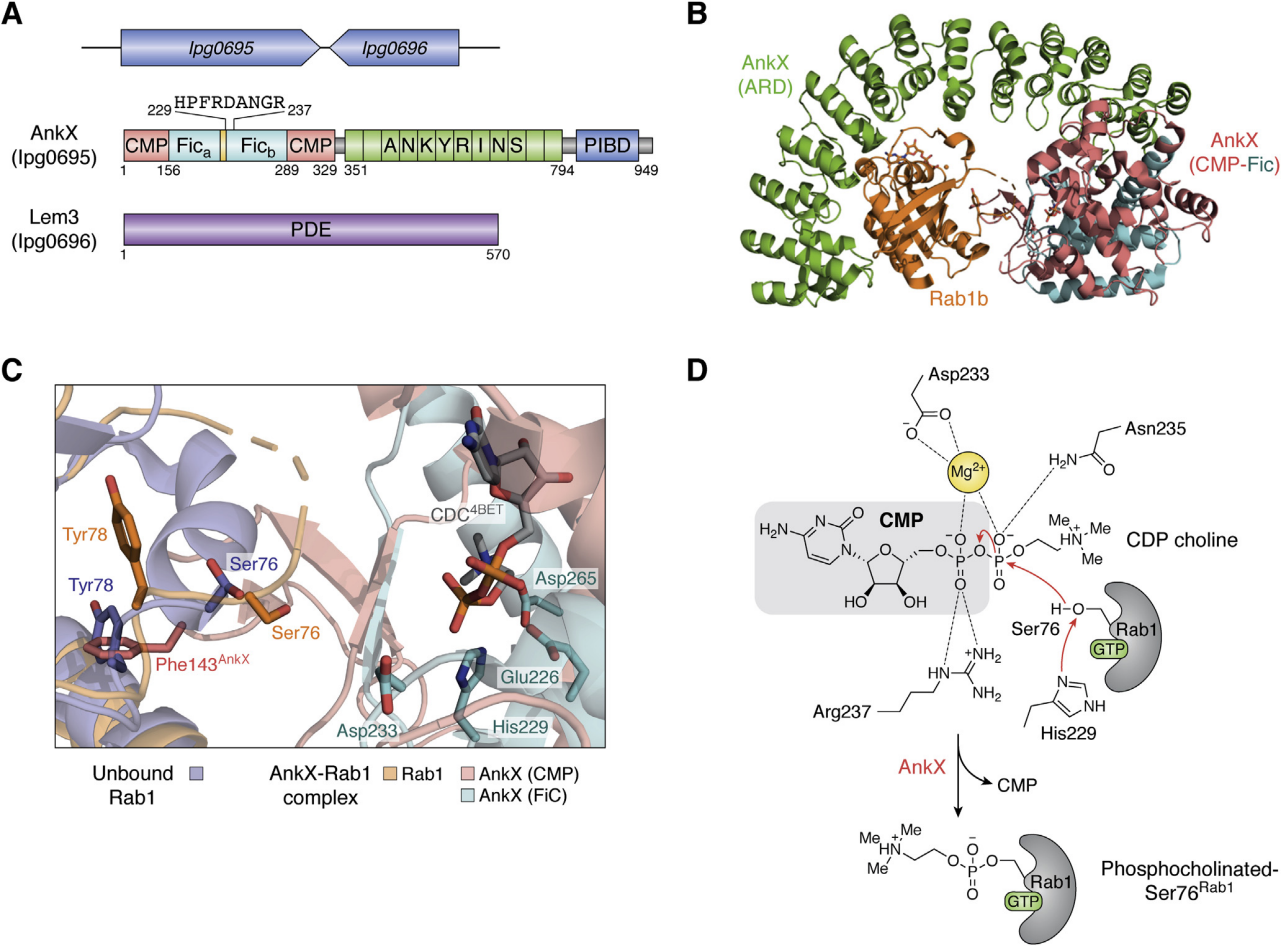
**Parallel modulators of Rab1 GTPase.***A*, Orientation of the genes, shown in *blue*, is indicated by the direction of the *arrows* they represent. The direction of both the genes is opposite to each other in the locus. Domains are labeled with the length of the proteins and colored individually. AnkX: CMP, N-terminal (cytidine monophosphate) binding domain (*red*); Fic_a/b_, filamentation induced by cAMP domain (*pale blue*), Ankyrins (*green*); PIBD, Phosphoinositide binding domain (*dark blue*). A unique 70-residue long insert (*yellow*) in the Fic domain is also shown. Functionally important residues from the Fic domain are shown. Lem3: PDE; phosphodiesterase domain (*purple*). *B*, crystal structure of AnkX-Rab1b complex (PDB code 6SKU). The different domains of AnkX are colored as per the domain diagram shown in Figure 6*A*. Rab1b is shown in *orange*. *C*, displacement of Switch II of Rab1b by AnkX. AnkX and Rab1b from the complex (PDB 6SKU) are colored as before. Superposed on Rab1b of this complex is the structure of unbound-Rab1b (PDB 3NKV) shown in *slate-blue*. The binding of AnkX to Rab1b displaces Switch II and locally unfolds the region adjacent to Ser76^Rab1b^, as can be seen when comparing the two structures. *D*, proposed catalytic mechanism of Rab1 phosphocholination mediated by AnkX. Asp233^AnkX^ (bound to the catalytic Mg^2+^) deprotonates His229^AnkX^, causing it to attack the scissile Oαβ-Pβ bond in CDP-choline and attach phosphocholine onto Ser76^Rab1^.

The AnkX and Lem3 effector pair represents a sophisticated example of functional redundancy used by *Legionella* to subvert the function of Rab GTPases and facilitate LCV maturation (Fig. 6*A*). A time-resolved analysis of *Legionella* effectors that modulate Rab1 function showed that these effectors differ in the specific timing of increase in their levels, which agrees with the role played by them in recruiting Rab1. Allombert *et al.* demonstrated that AnkX levels begin to rise only after SidM has been translocated and has had a chance to release Rab1 from the Rab1-GDI complex (101). AnkX subverts Rab1 (and Rab35) by functional (102) and structural (103, 104) mimicry of the Fic (filamentation induced by cAMP) domain, a domain known to catalyze AMPylation (105, 106). Proteins containing either a single or multiple Fic domains have been identified in all domains of life (107), most commonly in bacterial proteins, especially those involved in targeting host GTPases. The only known human protein containing this domain is the HypE protein, known to regulate protein stress response (105, 108, 109). The characteristic structural elements defining the Fic family of proteins are the presence of a bundle of six α-helices and a loop region with a highly conserved motif, HXFX(D/E)(A/G)N(G/K)R, the so-called Fic motif. The Fic domain of AnkX (Fig. 6*A*) catalyzes phosphocholination of Rab1 using CDP-choline, a modification that is ultimately reversed by Lem3 (110, 111). It resides within the CMP (cytidine monophosphate)-binding domain of AnkX and is unique because, unlike other Fic-domain-containing effectors, the Fic domain of AnkX transfers the phosphocholine moiety and not the nucleotide monophosphate. The C-terminal region of AnkX also contains ankyrin repeat domains (ARDs) and a PI3/4P-binding domain (Fig. 6*A*) (112). Akin to their role in eukaryotes, the ARDs in AnkX facilitate protein–protein interaction as observed between the ankyrin repeats 10 to 13 with the C-terminal of Rab1b in a crystal structure of the AnkX-Rab1b complex (104).

The different crystal structures of AnkX (in apo and Rab1-bound form) (Fig. 6*B*) revealed that the Fic domain plays a crucial role in binding the Switch I, II, and C-terminal regions of Rab1 (104, 113). This domain is divided into two subdomains separated by a unique 70-residue-long insert that masks the traditional binding site for GTPases on the Fic domain (Fig. 6, *A* and *B*). Phe107, Ile109, and Asp265 in the active site of AnkX sterically occlude the base of the CMP substrate from binding in the same orientation as seen with nucleotide substrates in other Fic enzymes. Instead, the choline group nestles in this region while the cytidine group stacks against Tyr41 of the CMP-binding domain, forcing a flipped orientation of the bound CMP moiety compared with the nucleotide-binding in AMPylating Fics. Thus, AnkX behaves as a phosphocholine transferase instead of a nucleotide transferase. Upon binding to AnkX, Switch II of Rab1b undergoes a significant structural rearrangement when Phe143^AnkX^ sticks into a hydrophobic pocket formed between Switch II and helix-3 of Rab1b. This encroachment by Phe143^AnkX^ displaces Tyr78^Rab1b^ from its highly conserved position in this hydrophobic cavity (Fig. 6*C*) (114). The displacement of Tyr78^Rab1b^ causes local unfolding of the Switch II region and the residues adjacent to it (Fig. 6*C*). As a result, the otherwise structurally restricted Ser76^Rab1^ of Switch II can now reach into the active site of AnkX for phosphocholination. The catalytic His229^AnkX^ of the Fic motif acts as a general base in deprotonating the OH group of Ser76^Rab1^, promoting a nucleophilic attack on the β-P center of the nucleotide (Fig. 6*D*). The Asp233 residue of the Fic motif positions the catalytic Mg^2+^, while the Asn235 and Arg237 play a critical role in interactions with the phosphocholine moiety.

The *Legionella* effector Lem3 (Fig. 6*A*), a phosphodiesterase whose structure is yet to be determined, can reverse the effects of AnkX by removing the phosphocholine group from Ser76 of Rab1 (110), making the GTPase accessible to other *Legionella* effectors such as LepB. Although phosphocholination on Ser76^Rab1^ does not affect the GEF activity of SidM, it does profoundly affect the adenylation of Tyr77^Rab1^, indicating that these modifications can be mutually exclusive. Ser76^Rab1^ modification by AnkX also negatively impacts the interactions of the modified Rab1 with its GDI, which are restored upon Lem3-catalyzed dephosphocholination of Rab1 (111). Surprisingly, Lem3 cannot hydrolyze AnkX-catalyzed phosphocholination of Rab35 on residue Thr76 (115) pointing to the existence of yet-to-be identified effector selective for the Rab35 modification. The importance of the AnkX/Lem3 pair in the hijacking of Rab1 is not as well understood as the role of the SidM/SidD pair. Nevertheless, given the importance of phosphocholination in the modulation of the eukaryotic immune system and the opposing activities of AnkX and Lem3, they are essential for the bacteria during infection.

The eight different activities mediated by seven *Legionella* effectors, SidM, AnkX, SidD, LepB, Lem3, SetA, and LidA (SetA and LidA have not been discussed here), known so far to modulate Rab1 function, point to the importance of the GTPase in the intracellular lifecycle of the bacteria. Upon activation, Rab1 interacts with other proteins, such as p115 or GM130, in order to guide ER-derived vesicles to fuse with the Golgi apparatus (116, 117, 118). Rab1 seems to play a similar role of docking ER-derived vesicles with the LCV during *Legionella* infection. Recruitment of Rab1 may also contribute to bypassing the default maturation of such organelles along the endocytic pathway for lysosomal degradation. SidM is translocated to the host cell within minutes of *Legionella* infection coinciding with Rab1 recruitment to the LCV (73, 101). It seems that once the GDP to GTP exchange occurs, mediated by SidM GEF domain, other effectors such as SetA, AnkX as well as the ATase domain of SidM are able to carry out specific PTMs targeting the Switch II residues Thr75, Ser76, and Tyr77, respectively (75, 110, 119). These mutually exclusive modifications might trap the activated Rab1 on the LCV membrane by preventing deactivation or dissociation by the eukaryotic GAPs and GDIs. On the other hand, detection of effectors such as SidD, LepB, and Lem3 in the later stages of infection suggests that these effectors temporarily control the recovery of Rab1 from the membrane by removing the modifications and deactivating the GTPase. Further studies are needed to reveal whether these effectors are specific for Rab1 alone or if they are responsible for modulating the broader landscape of GTPases, as indicated by their promiscuity toward several Rab proteins observed *in vitro* (75, 102).

## SidE proteins, SidJ and SdeD: atypical ubiquitination of Rab-GTPases

SidE family members, comprising SidE, SdeA, SdeB, and SdeC (8, 120), belong to yet another unique group of bacterial effectors that target several host proteins associated with the ER, including ER-Rabs (Rab1, for example) and reticulon, through phosphoribosyl-linked (PR-Ub) ubiquitination, a type of PTM for which there are no parallels in eukaryotes so far (28, 29, 49, 121, 122, 123). In contrast to the archetypical E1-E2-E3 three-enzyme system of eukaryotes, the SidE effectors use an all-in-one ubiquitinating machinery that utilizes NAD^+^, instead of ATP, to target serine residues of host proteins through PR-linked ubiquitination *via* Arg42 of Ub. This orthogonal mode of ubiquitination bypasses the eukaryotic machinery and produces a linker resistant to host DUBs (124).

**Figure 7:**
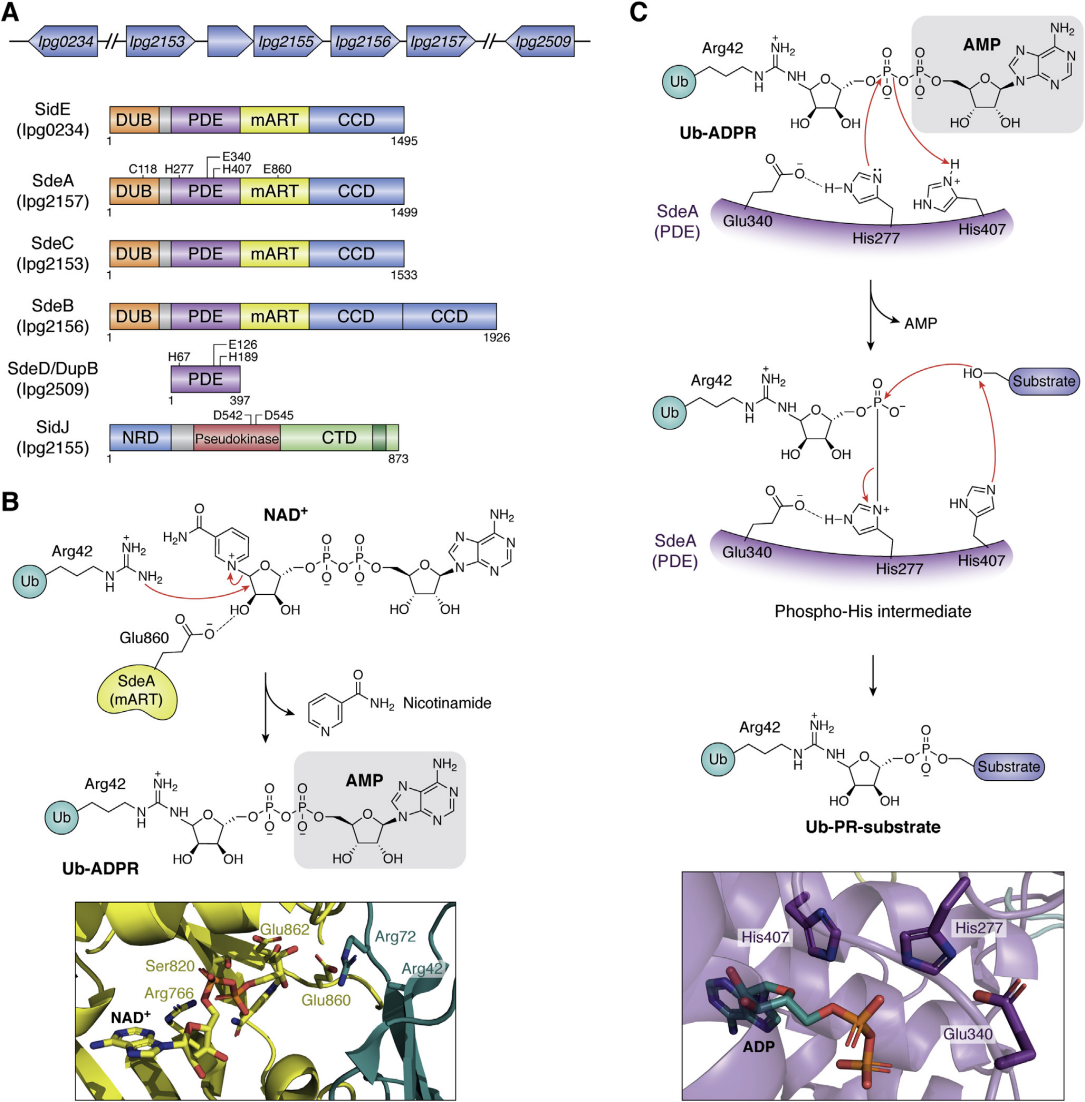
**SidE family membersand atypical ubiquitination.***A*, Orientation of the genes, shown in *blue*, is indicated by the direction of the *arrows* they represent. While *sdeA*, *sdeC*, *sdeB*, and *sidJ* belong to the same genetic locus, *sidE* and *sdeD* belong different loci. The domains of all the members are labeled with the length of the proteins. Different functional domains are colored individually: DUB, deubiquitinase domain (*orange*); PDE, phosphodiesterase (*purple*); mART, mono ADP-ribosyl transferase (*yellow*), and CCD, coiled-coil domain (*blue*). The functional domains in SidJ are NRD, N-terminal regulatory domain (*blue*), pseudokinase domain (*red*), and CTD, C-terminal domain (*pale green*). The IQ-motif where the eukaryotic protein, calmodulin, interacts with SidJ is colored *dark green*. Functionally important residues from each domain are shown for SdeA (the SidE family member discussed at length in this review). *B*, proposed catalytic mechanism of the mART domain. The catalytic Glu860^SedA^ facilitates ADP-ribosylation of Ub to generate an ADPR-Ub intermediate. This reaction utilizes NAD^+^ and releases nicotinamide. The *boxed panel* shows the SdeA-mART catalytic site in stick representation. Also shown are Arg72 and Arg42 (shown as Ala) from Ub. PDB codes: 5YSI, 5YIK. *C*, proposed catalytic mechanism of the PDE domain. The catalytic residues of the PDE domain cleave and transfer PR-Ub to a serine residue of a substrate protein. Reaction *arrows* are depicted in *red*. The SdeA-PDE catalytic site is shown in the *boxed panel*. PDB codes: 5YSI, 5YIK (127, 129).

SidE family members are large, functionally redundant proteins that share more than 40% sequence identity. These modular proteins function *via* the concerted action of four domains (Fig. 7*A*): a deubiquitinase domain (DUB), a phosphodiesterase domain (PDE), a mono-ADP-ribosyltransferase domain (mART), and a C-terminal coiled-coil domain (CC). Biochemical studies have shown that the DUB domain is not essential for the ubiquitinating activity of these proteins (125). SdeA likely uses its DUB domain to produce free Ub at the LCV to make it readily available for its ligase machinery. Dong *et al.* have shown that the CC domain interacts with parts of the Dot/Icm translocation machinery and may thus be required to inject the SidE ligases into the host cytosol (126). The core enzymatic machinery for ubiquitination comprises the PDE and the mART domains (126, 127, 128, 129). The mART domain, which features a characteristic RSE motif typically found in arginine-targeting mART enzymes, activates Ub by transferring the ADP-ribose group from NAD^+^ on to Arg42^Ub^, forming ADP-ribosylated Ub (Ub-ADPR) as an intermediate. Subsequently, the PDE domain catalyzes a phospho-transferase-like reaction where the PR-Ub of Ub-ADPR is transferred to the hydroxyl group of a serine residue of the host protein, accompanied by the release of AMP. Several structures of different constructs of these proteins (126, 127, 128, 129) (Table 1), along with biochemical studies, have allowed elucidation of some critical aspects of the various catalytic steps involved in recognition of Ub by the mART domain and those involved in recognition of Ub-ADPR by the PDE domain. The initial discovery of the five substrates: Rtn4 (reticulon 4) Rab1a, Rab6a, Rab30, and Rab33b (29, 122), was quickly followed by the understanding that the SidE proteins are tolerant of any serine that is a part of an unstructured/flexible region, provided it can be accommodated in the PDE active site (127, 130, 131). Since SidE proteins are known to colocalize with the LCV, it seems likely that the SidE enzymes target their substrates by proximity-based selection rather than by sequence specificity.

The mART domain of SdeA, like certain bacterial mART toxins, consists of a helical lobe and a main lobe, together forming the NAD^+^-binding pocket (132) at their interface. The nucleotide cofactor binds at this pocket in a strained conformation (Fig. 7*B*), which facilitates the departure of nicotinamide (Nic) prior to the attack of the Arg side chain on the resulting oxocarbenium center of the Nic-bearing ribose of NAD^+^ (126, 127). This reaction is similar to the one performed by bacterial mono-ADP-ribosyltransferase toxins, such as the Iota toxin from *Clostridium perfringens*, which utilizes the characteristic RSE active-site motif to ADP-ribosylate Arg177 of actin *via* the so-called SN1 strain alleviation mechanism of ADP-ribosylation (132). The conserved Arg and the Ser residues of SdeA help position and stabilize the strained conformation of the nucleotide in the active site, while the first Glu residue (Glu860 in SdeA) of the EXE dyad promotes the nucleophilic attack by the substrate Arg (Fig. 7*B*) and the second Glu stabilizes the oxocarbenium ion. The conformation of NAD^+^ observed in SidE mART crystal structures and the placement of critical residues of the RSE motif are consistent with the strain alleviation model of ADP-ribosylation in this enzyme.

The PDE domain of SdeA bears distinct sequence homology with other bacterial phosphodiesterases, such as the PDE domain of the well-known cyclic di-3′,5′-GMP phosphodiesterase PA4781 from *Pseudomonas aeruginosa* (133), with which it shares 23% sequence similarity. The PDE domain of SdeA has the same three catalytic residues, His277, Glu340, and His407, conserved in bacterial PDEs. Mechanistically, the reaction proceeds through covalent catalysis *via* the formation of a phospho-His-like intermediate (Fig. 7*C*) (28). His277 acts as a nucleophile to attack the β-phosphorous center of the ADPR moiety in Ub-ADPR, aided by Glu340 deprotonating the imidazole side chain to its neutral form (127). This attack results in the formation of a transient His277-PR-Ub intermediate and AMP release, enabled by proton donation by His407 to the leaving group. The His277-PR-Ub intermediate then reacts with the serine OH group of the substrate leading ultimately to the transfer of phosphoribosylated-ubiquitin (PR-Ub) onto the host protein (128, 130). From the position of His407 relative to the APDR moiety, it appears to be the most likely candidate for serving the role of the general base in activating the OH group of the target serine (Fig. 7*C*). Thus, despite the similarity of the PDE domain of SdeA with the bacterial phosphodiesterases, the catalytic motif in the SdeA PDE domain catalyzes a (substituted) phospho-transfer to a serine residue in the ubiquitination reaction instead of water, which would result in hydrolysis of the phosphodiester bond. Incidentally, the PDE domain of the SdeA can also catalyze phospho-transfer to water, ensuing from water attacking the phospho-His intermediate, resulting in the PR-Ub hydrolysis product (130). The biological significance of this side reaction in the context of *L. pneumophila* infection remains to be determined, as PR-Ub can be toxic to host cells when the SidE members are ectopically expressed in mammalian cells.

Even though the SidE proteins are essential for *Legionella* replication in eukaryotes, unchecked activity of these proteins may result in the accumulation of free PR-Ub that would inhibit the host ubiquitination machinery (28). This contamination of the cellular Ub pool leads to impairment of crucial Ub-dependent cellular processes (28). Ubiquitination of substrates such as Rab1 by members of the SidE family may also affect the activity of other effectors such as SidM and AnkX that need Rab1. The PR-ubiquitination of host proteins triggered by the SidE family members is regulated by two effectors: SidJ and SdeD. *sidJ* resides in the same genetic locus as *sdeC*, *sdeB*, and *sdeA*, whereas *sdeD* is located much further down in a distinct genomic locus (Fig. 7*A*) (134, 135). Studies have shown that SidJ can inactivate SidE proteins by directly inhibiting the mART activity, thereby shutting off the ubiquitination reaction, whereas SdeD, also known as DupB (deubiquitinase of phosphor-ribosyl linked ubiquitination), and its paralog DupA, can counterbalance the activity of the SidE members by acting on the phosphoribosylated host proteins (125, 128, 136, 137).

**Figure 8:**
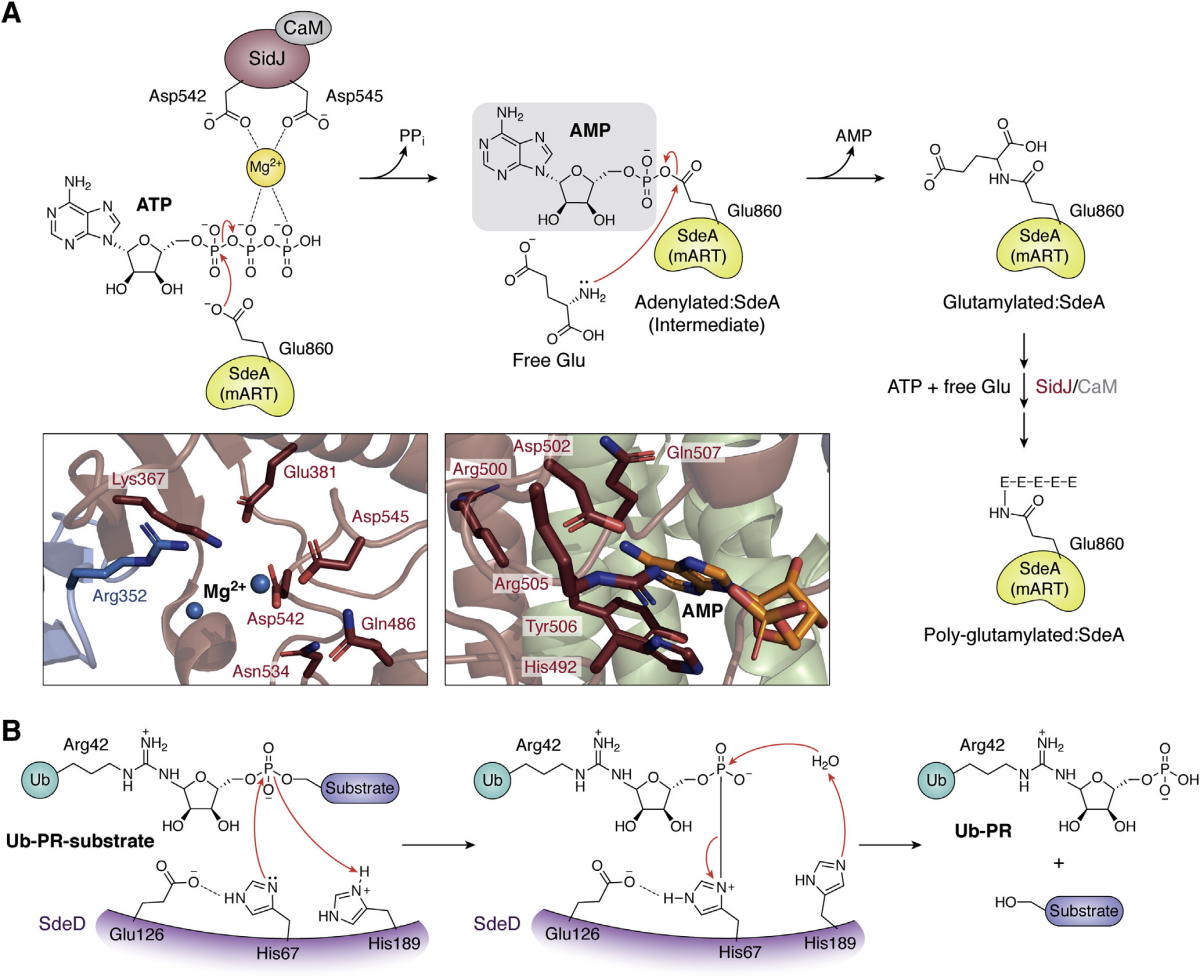
**Regulation of the activity of SidE members by SidJ and SdeD.***A*, proposed catalytic mechanism of SdeA glutamylation catalyzed by SidJ. In the presence of ATP and Mg^2+^, the kinase-like domain of SidJ transfers the AMP group onto the catalytic Glu860^SdeA^, forming an adenylated-SdeA intermediate. A free glutamate then attacks this intermediate to covalently attach the glutamate residue onto Glu860^SdeA^. Also shown in the *boxed panels* are the residues that form the two putative active sites in SidJ. *B*, proposed catalytic mechanism of SdeD. H67^SdeD^ cleaves the β-phosphate of the ADPR to form an intermediate linking H67^SdeD^ to the phosphate of PR-Ub. H189^SdeD^ then abstracts a proton from the incoming water molecule to facilitate its attack on the phosphate, resulting in the formation of Ub-PR and free substrate.

SidJ is an 873 amino-acid protein expressed in the later stages of *Legionella* infection. Inhibition of SidE proteins by SidJ is both temporally regulated and spatially restricted as it requires the host calmodulin (CaM) for its activity (138, 139). Compartmentalization of SidJ's activity in the host cytosol prevents premature inactivation of SidE effectors before being injected into the host cell. Biochemical studies have revealed that the association of SidJ with CaM stabilizes the active conformation of the effector (138, 139, 140, 141, 142). Structure–function studies (138, 139, 140, 141) revealed SidJ to be a pseudokinase that utilizes ATP to catalyze the polyglutamylation of SdeA (and other SidE members). CaM binding *via* the IQ motif located at its C-terminal end of SidJ presumably opens the kinase-like active site of SidJ for ATP. In the first step of catalysis, SidJ uses ATP to acyl-adenylate the carboxylate group of SdeA Glu860 with the release of pyrophosphate (Fig. 8*A*), a reaction akin to the acyl-adenylation step of the reaction catalyzed by aminoacyl-tRNA synthetases or activation of Ub by E1. The activated carbonyl of this unstable intermediate is then attacked by the amino group of a free glutamate residue, leading to glutamylation of the SdeA catalytic residue *via* an isopeptide linkage and the release of AMP (Fig. 8*A*). This second step likely involves another essential region in SidJ, named the “migrated” nucleotide-binding site (139), which may help in the optimal positioning of the acyl-adenylated SdeA and the free glutamate to take the reaction to completion. A recent cryo-EM reconstruction of the SidJ:CaM:SdeA intermediate complex revealed that while the pseudokinase active site is responsible for the acyl-adenylation reaction, it is the migrated nucleotide binding pocket that carries out the glutamylation reaction, with Arg522^SidJ^ playing the crucial role of positioning the donor Glu to attack the acyl-adenylate intermediate and subsequent formation of the Glu-Glu isopeptide bond on SdeA (143).

SdeD (DupB) shares overall structural similarity with the PDE domain of the SidE proteins (127, 130) along with a highly conserved catalytic core made up of His67, Glu126, and His189 (His277, Glu340, and His407 in SdeA-PDE domain) (Fig. 7*A*). SdeD acts as a hydrolase to catalyze the hydrolysis of PR-ubiquitinated substrates, presumably proceeding *via* the formation of a histidine-based phosphoramidate intermediate such as the SdeA-PDE mechanism (136) (Fig. 8*B*). SdeD can remove the AMP moiety from Ub-ADPR, like the hydrolysis reaction catalyzed by the PDE domain of SidE members. However, it cannot transfer the PR-Ub onto target proteins, likely due to the structural differences that alter the conformation and accessibility to the catalytic center in the two effectors. The SdeD loop comprising residues 26 to 48 is missing in SdeA, and conversely, the SdeA loop comprising residues 465 to 513 does not exist in SdeD. It is possible that because of this structural difference, in SdeD, only water can act as the ubiquitin acceptor instead of the hydroxyl group of a serine residue (Fig. 8*B*) (137). The SdeA-PDE domain and SdeD pair is another example, much like the MavC-MvcA pair discussed above, of *Legionella* adopting the same catalytic machinery to mediate opposite reactions.

The ligase activity of SidE members results in serine ubiquitination of several structurally diverse substrates, the consequences of which affect a variety of cellular processes, ranging from autophagy and vesicular trafficking to tubular ER dynamics and inhibition of protein synthesis. It appears that the seemingly indiscriminate nature of the SidE proteins contributes to the virulence of the pathogen and enables the establishment of the LCV. For example, by PR-ubiquitinating Rab6a and Rab33b, these effectors modulate the Golgi-to-ER retrograde trafficking and prevent the formation of autophagosomes ((144) and all the references therein), an essential first step in autophagy. Similarly, by ubiquitinating Rag GTPases, the SidE effectors inactivate mammalian target of rapamycin complex 1 (mTORC1) to inhibit host protein synthesis (145), effectively allowing the pathogen to consume the host amino acids as nutrients for its survival. It is believed that PR-ubiquitination by the SidE members is most likely regulated at the early stages of infection by SdeD to prevent unchecked accumulation of Ub-ADPR and depletion of cellular Ub (137). In contrast, SidJ regulates the removal of SidE proteins from the LCV at later stages of infection (125, 135). However, despite understanding the biochemistry of the reactions mediated by these effectors, it is not yet clear whether these activities are exerted simultaneously or if they are a cascading consequence of one another. Further studies are required to fully appreciate the spatiotemporal regulation of cellular processes modulated by them.

## Other examples of effector–effector pairs

Identification of effector–effector interactions is often the consequence of studying the function of an individual effector. However, recently, studies have systematically explored the interactions between effector pairs by combining a gain-of-function genetic screen in yeast with cellular and biochemical approaches. Urbanus *et al*. (146) successfully identified novel, direct pairwise effector–effector interactions by carrying out a comprehensive analysis of all possible pairwise interactions between the 330 effectors secreted by *L. pneumophila*. Pairwise interactions that merit a mention even though there is not enough structural information available at present to describe their mechanism of action in detail are (1) inactivation of RavJ by LegL1 (2), polyubiquitination of SidH by LubX, and (3) inhibition of MavQ by SidP.

RavJ, a small *Legionella* effector, rearranges the actin cytoskeleton leading to the accumulation of more F-actin on the plasma membrane (147). It consists of two domains: An N-terminal papain-like cysteine protease domain and a C-terminal domain that interacts with various cytoskeleton-associated components of the eukaryotic septin and elongator complexes (146). Many pathogenic bacteria have evolved virulence factors that specifically target Rho GTPases, which control the reorganization of the actin cytoskeleton (148). For example, YopT, an effector protein from *Yersinia*, functions as a cysteine protease to cleave Rho GTPases and inhibits phagocytosis by disrupting the actin cytoskeleton. The N-terminal domain of RavJ has the requisite active-site elements that can potentially disrupt the actin cytoskeleton such as YopT, although the specific catalytic activity remains to be demonstrated. LegL1 is a leucine-rich repeat (LRR)-containing *Legionella* effector that binds RavJ (146) and inhibits its activity by blocking the putative active site. It is unclear at which point, postinfection, RavJ and LegL1 are employed during the intracellular life cycle of *L. pneumophila* and temporal regulation mediated by the effector–effector interaction.

Historically, it is the discovery of the SidH-LubX effector pair that first brought to light that *Legionella* effectors can regulate the activity of each other. LubX is a U-box containing bacterial E3 ligase (149) that can polyubiquitinate SidH (150), thereby targeting it to the proteasome for degradation. Initial pull-down experiments revealed that of the U-box motifs present in LubX U-box2 (150) mediates the physical interaction between the two effectors, while *in vitro* ubiquitination assays and structural studies showed that the U-box1 is responsible for the polyubiquitination property of the effector *via* recruitment of the host Ub-conjugating E2 enzyme, Ube2d3 (150, 151). The function of SidH within the host is yet unclear. However, it is believed that it may contribute to the maintenance of the integrity of the LCV like its paralog SdhA (150, 152). Pfam analysis of the SidH sequence identifies two potential functional motifs: the I_LWEQ motif that signifies binding to F-actin and a polysaccharide deacetylase motif (KEGG database; (57)). The presence of these motifs in SidH and its appearance in the early stages of *Legionella* infection indicate that perhaps SidH functions to regulate cell surface dynamics *via* these motifs. Further structure–function studies are required to understand the functional importance of SidH and its inhibition by LubX.

The partnering between a phosphatase (SidP) and a kinase (MavQ) is involved in phosphatidylinositol polyphosphate (PIP) modulation. The amino acid sequence of SidP bears no homology to any known phosphatidylinositol phosphatase (PI phosphatase), while the MavQ sequence is an atypical kinase. Biochemically, SidP is similar to the CX5R-based PI phosphatases that belong to the myotubularin family (153, 154) in that it cannot hydrolyze PI species with two adjacent phosphate groups such as PI(3,4) (155) but is able to hydrolyze PI(3)P and PI(3,5)P2. SidP inhibits MavQ by binding to its C-terminal domain, indicating that the PI phosphatase activity, which resides in the N-terminal domain of SidP, is distinct from its role of MavQ inhibition. A recent study by Hsieh *et al.* (156) showed that MavQ and SidP work alongside each other, the former adding a phosphate to PI(3)P moieties and the latter removing phosphates from higher-order phospholipids or even converting PI(3)P to simple PI entities and altering the lipid composition of the host membrane, in the context of infection.

## *Legionella* effectors and cellular homeostasis

**Figure 9:**
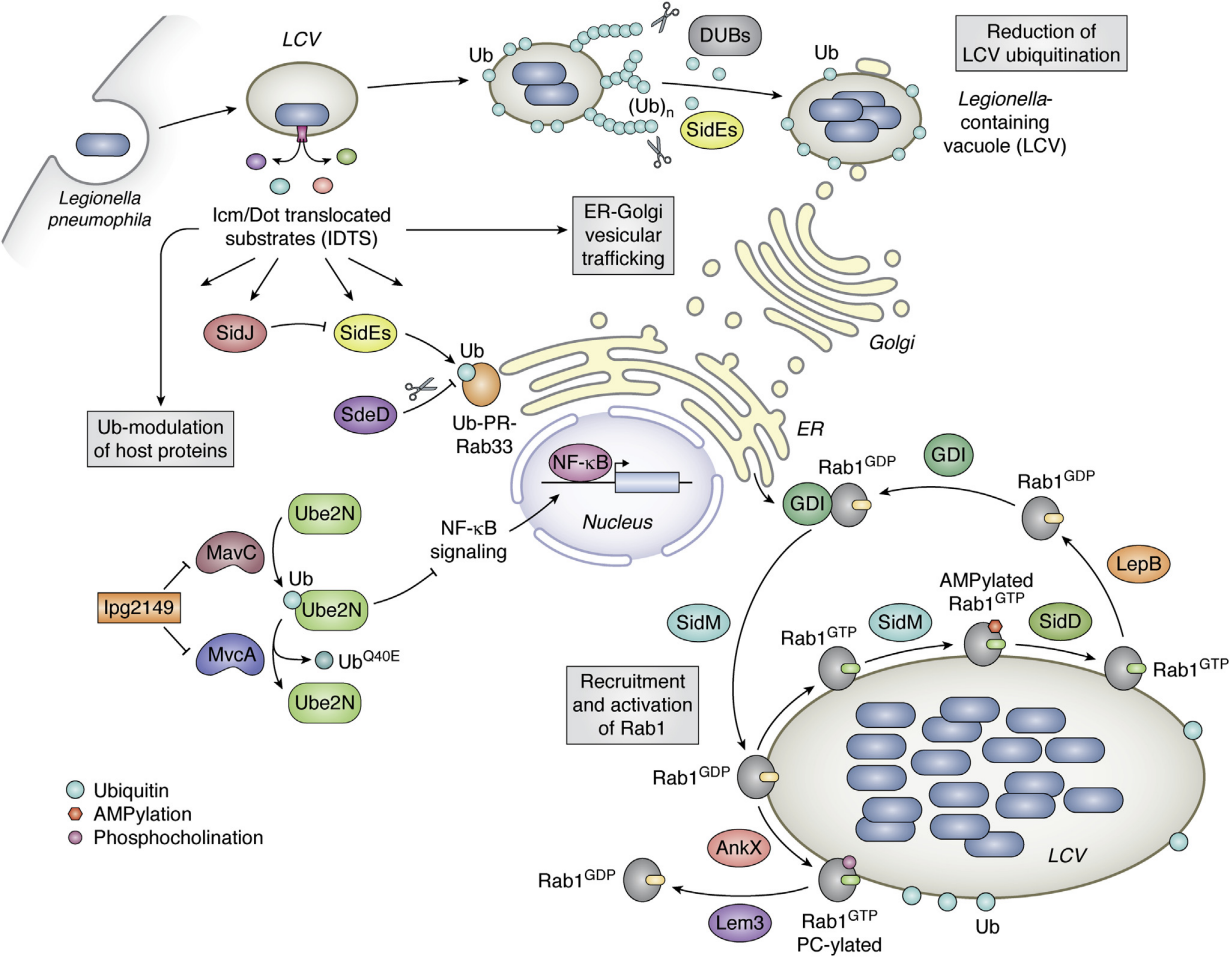
**Coordinated regulation of different host cellular pathways by *Legionella pneumophila* effectors.** Upon bacterial infection, Ub-modulating effectors of the pathogen interfere with the vesicular trafficking between the ER and the Golgi to establish the Legionella-containing vacuole (LCV). SidE proteins PR-ubiquitinate several host proteins, including Rab33, SdeD removes this modification from the substrates, and SidJ negatively regulates the activity of the SidE proteins. The bacterial DUBs act to reduce ubiquitination on the LCV. SidE proteins can also serve to remove Ub from the LCV. Soon after infection, the bacteria target the ER-associated GTPase, Rab1. SidM recruits Rab1 to the LCV and activates it. PTMs such as AMPylation (by SidM) and phosphocholination (by AnkX) lock Rab1 in its active conformation (Rab1GTP). At later stages of infection, SidD and Lem3 remove these modifications from Rab1, allowing it to be inactivated by LepB. Rab1GDP is then removed from the LCV by GDIs. *Legionella* infection also affects the host immune response. Ubiquitination of Ube2N by MavC interferes with Lys63-linked polyubiquitination, dampening the NF-κB signaling pathway. MvcA and lpg2149 counteract the ubiquitinating activity of MavC at later stages of infection.

*L*. *pneumophila* allocates about 10% of its protein-coding ability toward functions that require direct interactions with host cellular processes. One of the most remarkable features of *Legionella* is its capacity to both temporally and spatially regulate the dynamics of its effectors during infection. Three regulatory systems (the PmrAB two-component system, the CpxRA two-component system, and the LetAS-RsmYZ-CsrA regulatory cascade) directly or indirectly regulate the expression of several effector-encoding genes (157, 158, 159, 160, 161). These regulatory systems work cohesively to allow the pathogen to enter the host cell, adapt to the new environment, and regulate host mechanisms that promote multiplication and survival within the cell. For example, *Legionella* triggers the nuclear localization of NF-κB in macrophages to positively upregulate antiapoptotic genes in a Dot/Icm-dependent manner to support intracellular bacterial growth (56). Another essential cellular machinery targeted by *L. pneumophila* is the host amino acid metabolism regulated by mTORC1. As alluded to before, the pathogen frees up host amino acids for its consumption *via* a concerted albeit temporally regulated action of SidE and Lgt family of effectors (145). Another well-studied example is the co-option of the host ubiquitin network by *Legionella*. On the one hand, the translocated effectors such as MavC and SidE proteins ligate Ub onto host proteins, inactivating or altering their function in the process (Fig. 9). On the other hand, ubiquitination is also used as a targeting signal for other effectors, as exemplified by the negative regulation of PR-ubiquitination by SidJ.

## Conclusions

It is not surprising that there is a constant battle between pathogens and their hosts to develop means to adapt and counter-adapt during evolution. In contrast to the high conservation of the secretion system itself among different *Legionella* species, the effector arsenal is quite varied, suggesting that *Legionella*'s genomic flexibility is because of its coevolution with numerous protists species that belong to the phyla Amoebozoa and Percolozoa (162). *Legionella* effectors can regulate a myriad of cellular functions (Fig. 9) because the bacteria may have acquired numerous genes from a range of primitive eukaryotes through horizontal gene transfer during evolution (162). This unique eukaryotic-like repository of effectors results from the exogenous acquisition of numerous eukaryotic domains that function as fundamental building blocks (163). These building blocks can be rearranged to generate new domain/motif combinations, contributing to the evolution of this unexpectedly large arsenal of functionally diverse and seemingly redundant effectors. Despite a high rate of evolution over long periods, the amino acids involved in protein–protein interaction have undergone positive selection pressure (164, 165). This suggests that sometimes despite low overall sequence homology between bacterial proteins and their eukaryotic counterparts, the residues at protein interfaces are well conserved, pointing to an essential role for bacterial effectors in interfering with host pathways (Fig. 9).

Many *L. pneumophila* effectors have been identified using various genetic and biochemical techniques. However, it is still unclear why the pathogen requires so many effectors for its survival within its hosts. Perhaps it is to ensure sustained intracellular replication and is simply an indication of the numerous eukaryotic pathways regulated by the bacteria (Fig. 9), making the study of these effectors quite complex, albeit exciting. Our understanding of bacterial virulence and its impact on host signaling is hampered by the built-in functional redundancy exhibited by many of these effectors. The recent discovery of functional interplay between bacterial proteins to modulate each other has given rise to the concept of effector–effector synergism that adds another intriguing dimension to the various modes of survival adopted by *Legionella*. Despite the diverse nature of the different eukaryotic functions targeted by these effectors, one emerging theme is the existence of a yin and yang type of mechanism. It would not be unreasonable to assume that these principles are co-opted by pathogens in general and not just by bacteria. Future structure–function studies will not only help in evaluating the role of pathogenic effectors but will also aid us in understanding how essential eukaryotic cellular homeostasis is maintained.

## Conflict of interest

The authors declare that they have no conflict of interest with the contents of this article.
